# Supplementary material for: Novel Quantitative PCR for Rhodococcus equi and Macrolide Resistance Detection in Equine Respiratory Samples
Source: Animals (Basel). 2022 May 3;12(9):1172. doi: 10.3390/ani12091172 (PMC9099730; doi:10.3390/ani12091172)
Supplement: Supplementary file 1 [file animals-12-01172-s001.zip › animals-1664336-supplementary.pdf]

Table S1. List of *R. equi* genomes included in the in-silico validation

Genetic element	Strain	Isolation source	Macrolide susceptibility	GenBank no.
WGS	lh_4_1	Environment	S	WUZO00000000
WGS	lh_25_1	Environment	S	WVAD00000000
WGS	lh_27_1	Environment	S	WVAA00000000
WGS	lh_29_1	Environment	S	WUZY00000000
WGS	lh_31_1	Environment	S	WUZV00000000
WGS	lh_33_1	Environment	S	WUZU00000000
WGS	lh_35_1	Environment	S	WUZS00000000
WGS	lh_37_1	Environment	S	WUZQ00000000
WGS	lh_39_1	Environment	S	WUZR00000000
WGS	lh_48_1	Environment	S	WUZH00000000
WGS	lh_54_1	Environment	S	WUYZ00000000
WGS	lh_57_1	Environment	S	WVDI00000000
WGS	lh_63_1	Environment	S	WVDD00000000
WGS	lh_65_1	Environment	S	WVDA00000000
WGS	lh_72_1	Environment	S	WVCU00000000
WGS	lh_11_1	Environment	S	SAMN13391722
WGS	lh_84_1	Environment	S	WVCJ00000000
WGS	lh_93_1	Environment	S	SAMN13391754
WGS	lh_96_1	Environment	S	WVDW00000000
WGS	lh_13_1	Environment	S	SAMN13391723
WGS	lh_108_1	Environment	S	WVBV00000000
WGS	lh_111_1	Environment	S	SAMN13391760
WGS	lh_42_1	Environment	S	WUZL00000000
WGS	lh_117_1	Environment	S	WVBM00000000
WGS	lh_120_1	Environment	S	WVBK00000000
WGS	lh_123_1	Environment	S	WVBJ00000000
WGS	lh_126_1	Environment	S	WVBC00000000
WGS	lh_129_1	Environment	S	SAMN13391766
WGS	lh_132_1	Environment	S	WVAY00000000
WGS	lh_135_1	Environment	S	WVAV00000000
WGS	lh_138_1	Environment	S	SAMN13391769
WGS	lh_141_1	Environment	S	WVAR00000000
WGS	lh_144_1	Environment	S	WVAN00000000
WGS	lh_42_1	Environment	S	SAMN13391737
WGS	lh_69_1	Environment	S	SAMN13391746
WGS	lh_1_1	Environment	R	WVCG00000000
WGS	lh_20_1	Environment	R	WVAH00000000
WGS	lh_22_1	Environment	R	WVAF00000000
WGS	lh_24_1	Environment	R	WVAE00000000
WGS	lh_26_1	Environment	R	WVAB00000000
WGS	lh_28_1	Environment	R	WUZZ00000000
WGS	lh_30_1	Environment	R	WUZW00000000
WGS	lh_32_1	Environment	R	SAMN13391644
WGS	lh_34_1	Environment	R	WUZT00000000
WGS	lh_36_1	Environment	R	WUZP00000000
WGS	lh_41_1	Environment	R	WUZM00000000
WGS	lh_55_1	Environment	R	WVCB00000000
WGS	lh_58_1	Environment	R	WVDJ00000000
WGS	lh_61_1	Environment	R	WVDF00000000
WGS	lh_64_1	Environment	R	WVDC00000000
WGS	lh_67_1	Environment	R	WVDB00000000
WGS	lh_70_1	Environment	R	SAMN13391667
WGS	lh_8_1	Environment	R	WVCN00000000
WGS	lh_73_1	Environment	R	WVCS00000000
WGS	lh_79_1	Environment	R	WVCQ00000000

WGS	lh_82_1	Environment	R	WVCL00000000
WGS	lh_85_1	Environment	R	WVCI00000000
WGS	lh_88_1	Environment	R	WVCF00000000
WGS	lh_91_1	Environment	R	WVEB00000000
WGS	lh_97_1	Environment	R	SAMN13391685
WGS	lh_100_1	Environment	R	SAMN13391687
WGS	lh_9_1	Environment	R	WVEI00000000
WGS	lh_106_1	Environment	R	WVBW00000000
WGS	lh_109_1	Environment	R	WVBU00000000
WGS	lh_112_1	Environment	R	WVBR00000000
WGS	lh_115_1	Environment	R	WVBQ00000000
WGS	lh_118_1	Environment	R	WVBN00000000
WGS	lh_124_1	Environment	R	SAMN13391703
WGS	lh_127_1	Environment	R	WVBE00000000
WGS	lh_130_1	Environment	R	WVBB00000000
WGS	lh_10_1	Environment	R	WVCD00000000
WGS	lh_133_1	Environment	R	WVAZ00000000
WGS	lh_136_1	Environment	R	WVAW00000000
WGS	lh_12_1	Environment	R	WVBL00000000
WGS	lh_14_1	Environment	R	WVAS00000000
WGS	lh_16_1	Environment	R	WVAM00000000
WGS	lh_18_1	Environment	R	WVAK00000000
WGS	lh_2_1	Environment	R	WVAI00000000
WGS	lh_6_1	Environment	R	WVDG00000000
WGS	lh_47_1	Environment	R	WUZF00000000
WGS	lh_50_1	Environment	R	WUZD00000000
WGS	lh_53_1	Environment	R	WUZA00000000
WGS	lh_56_1	Environment	R	WVCC00000000
WGS	lh_62_1	Environment	R	WVDE00000000
WGS	lh_65_1	Environment	R	WVCY00000000
WGS	lh_68_1	Environment	R	WVCX00000000
WGS	lh_71_1	Environment	R	WVCW00000000
WGS	lh_74_1	Environment	R	SAMN13391670
WGS	lh_80_1	Environment	R	WVCO00000000
WGS	lh_83_1	Environment	R	WVCK00000000
WGS	lh_86_1	Environment	R	WVCE00000000
WGS	lh_89_1	Environment	R	WVDY00000000
WGS	lh_92_1	Environment	R	SAMN13391682
WGS	lh_95_1	Environment	R	WVDP00000000
WGS	lh_98_1	Environment	R	WVEL00000000
WGS	lh_101_1	Environment	R	SAMN13391688
WGS	lh_104_1	Environment	R	WVBY00000000
WGS	lh_113_1	Environment	R	WVBS00000000
WGS	lh_116_1	Environment	R	WVBO00000000
WGS	lh_119_1	Environment	R	SAMN13391700
WGS	lh_122_1	Environment	R	WVBI00000000
WGS	lh_125_1	Environment	R	WVBG00000000
WGS	lh_128_1	Environment	R	WVBD00000000
WGS	lh_131_1	Environment	R	WVBA00000000
WGS	lh_137_1	Environment	R	SAMN13391712
WGS	lh_140_1	Environment	R	WVAT00000000
WGS	lh_16	Horse	R	WUXS00000000
WGS	lh_17	Horse	R	WUXR00000000
WGS	lh_18	Horse	R	WUXQ00000000
WGS	lh_19	Horse	R	WUXP00000000
WGS	lh_20	Horse	R	WUXO00000000
WGS	lh_21	Horse	R	WUYT00000000
WGS	lh_24	Horse	R	WUXL00000000
WGS	lh_25	Horse	R	WUXK00000000

WGS	lh_26	Horse	R	WUXJ00000000
WGS	lh_27	Horse	R	WUYS00000000
WGS	lh_28	Horse	R	WUXI00000000
WGS	lh_29	Horse	R	WUXH00000000
WGS	lh_30	Horse	R	WUXG00000000
WGS	lh_32	Horse	R	WUYR00000000
WGS	lh_34	Horse	R	WUYQ00000000
WGS	lh_36	Horse	R	WUYO00000000
WGS	lh_37	Horse	R	WUYN00000000
WGS	lh_39	Horse	R	WUXC00000000
WGS	lh_40	Horse	R	WUYM00000000
WGS	lh_41	Horse	R	WUYL00000000
WGS	lh_43	Horse	R	WUXA00000000
WGS	lh_44	Horse	R	WUYK00000000
WGS	lh_45	Horse	R	WUWZ00000000
WGS	lh_47	Horse	R	WUYJ00000000
WGS	lh_48	Horse	R	WUYI00000000
WGS	lh_50	Horse	R	WUYH00000000
WGS	lh_8	Horse	R	WUXZ00000000
WGS	PAM2275	Horse	R	MULU01000000
WGS	PAM2277	Horse	R	MUMB01000000
WGS	PAM2280	Horse	R	MULW01000000
WGS	PAM2281	Horse	R	MULT01000000
WGS	PAM2283	Horse	R	MULY01000000
WGS	PAM2284	Horse	R	MULZ01000000
WGS	PAM2285	Horse	R	LWTU00000000
WGS	PAM2286	Horse	R	MULX01000000
WGS	PAM2287	Horse	R	LWTV00000000
WGS	PAM2289	Horse	R	MUXK01000000
WGS	PAM2292	Horse	R	MVDT01000000
WGS	PAM2293	Horse	R	MVDU01000000
WGS	PAM2294	Horse	R	MVDV01000000
WGS	PAM2295	Horse	R	MVDQ01000000
WGS	PAM2296	Horse	R	MVDR01000000
WGS	PAM2297	Horse	R	MUXJ01000000
WGS	lh_1	Horse	S	WUYG00000000
WGS	lh_3	Horse	S	WUYE00000000
WGS	lh_5	Horse	S	WUYC00000000
WGS	lh_6	Horse	S	WUYB00000000
WGS	lh_7	Horse	S	WUYA00000000
WGS	lh_9	Horse	S	SAMN13392186
WGS	lh_13	Horse	S	WUXV00000000
WGS	lh_14	Horse	S	WUXU00000000
WGS	lh_15	Horse	S	WUXT00000000
WGS	lh_22	Horse	S	WUXN00000000
WGS	lh_31	Horse	S	WUXF00000000
WGS	lh_33	Horse	S	WUXE00000000
WGS	lh_35	Horse	S	WUYP00000000
WGS	lh_38	Horse	S	WUXD00000000
WGS	lh_42	Horse	S	WUXB00000000
WGS	lh_46	Horse	S	WUWY00000000
WGS	PAM2274	Horse	S	LWTQ00000000
WGS	PAM2276	Horse	S	LWTR00000000
WGS	PAM2282	Horse	S	LWTT00000000
WGS	N1301	Environment	S	LRRA00000000
WGS	ATCC 13557	-	S	JAFFSZ0100000
WGS	ATCC 33707	-	S	ADNW00000000
Complete	FDAARGOS_952	-	S	CP065594

WGS	DE0128	Environment	S	VEFT01000000
WGS	DE0411	Environment	S	VDTB01000000
WGS	DSM 20307	Horse	S	LWTX00000000
WGS	DSM 20295	Environment	S	LRRF00000000
Complete	DSSKP-R-001	Environment	S	CP027793,CP027794,CP027795
WGS	N1288	Pig	S	LRQY00000000
WGS	N1295	Horse	S	LRQZ00000000
Complete	103S	Horse	S	NC_014659
WGS	NCTC1621	-	S	UGVJ01000000
WGS	NCTC5650	-	S	UGVG01000000
WGS	PAM1204	Sheep	S	LWBN00000000
WGS	PAM1216	Horse	S	LWHS00000000
WGS	PAM1271	Horse	S	LWIC00000000
WGS	PAM1340	Horse	S	LWHT00000000
WGS	PAM1354	Human	S	LWHU00000000
WGS	PAM1357	Horse	S	LWHV00000000
WGS	PAM1413	Human	S	LWHW00000000
WGS	PAM1422	Horse	S	LWHX00000000
WGS	PAM1475	Pig	S	LWHY00000000
WGS	PAM1496	Pig	S	LWHZ00000000
WGS	PAM1533	Pig	S	LWIA00000000
WGS	PAM1557	Cow	S	LWIB00000000
WGS	PAM1571	Cow	S	LWTO00000000
WGS	PAM1572	Cow	S	LXFI00000000
WGS	PAM1593	Human	S	LXFH00000000
WGS	PAM1600	Horse	S	LXFG00000000
WGS	PAM1637	Horse	S	LWHR00000000
WGS	PAM1643	Horse	S	LWTP00000000
WGS	PAM2012	Cow	S	LWTY00000000
WGS	UBA6654	Environment	S	DKKY01000000
Complete	WY	Human	S	CP041647,CP041646

Table S2. In the in-silico validation of oligos and probes in 25 non-*R.equi* bacterial genomes (chromosomes and plasmids)

		Match with Rhodo_Dlab			Match with Erm46_Dlab			Match with Erm51_Dlab		
**Species chromosome**	**RefSeq no.**	**FW**	**RV**	**Probe**	**FW**	**RV**	**Probe**	**FW**	**RV**	**Probe**
Rhodococcus triatomae strain DSM 44,893	GCF_014217765.1	No	No	No	No	No	No	No	No	Yes (8)
Rhodococcus defluvii strain Ca11	GCF_000738775.1	Yes (0)	No	No	No	Yes (4)	Yes (7)	No	No	Yes (8)*
Rhodococcus ruber strain C1	GCF_016804345.1	No	No	No	No	No	No	No	No	Yes (6)
Rhodococcus rhodochrous strain EP4	GCF_003004765.2	No	No	No	No	No	No	No	No	Yes (4)
Rhodococcus qingshengii strain CS98	GCF_015099595.1	No	Yes (1)	No	No	No	No	No	No	No
Rhodococcus pyridinivorans strain SB3094	GCF_000511305.1	No	No	No	No	No	No	No	No	Yes (6)
Rhodococcus opacus strain 1CP	GCF_001685605.1	No	No	No	No	No	No	No	No	Yes (6)
Rhodococcus jostii strain DSM 44719	GCF_900105375.1	No	No	No	No	Yes (5)	Yes (7)	No	No	No
Rhodococcus fascians strain D188	GCF_001620305.1	No	No	No	No	No	Yes (7)	No	No	No
Rhodococcus erythropolis strain R138	GCF_000696675.2	No	Yes (0)	No	No	No	No	No	No	No
Mannheimia haemolytica strain USDA-ARS-USMARC-191	GCF_002285575.1	No	No	No	No	Yes (5)	No	No	No	No
Nocardia asteroides strain NCTC11293	GCF_900637185.1	No	Yes (1)	No	No	No	No	No	No	No
Mycolicibacterium smegmatis strain FDAARGOS_679	GCF_013349145.1	No	Yes (2)	No	No	No	No	No	No	No
Bordetella bronchiseptica strain NCTC10543	GCA_900636925.1	No	Yes (1)	No	No	No	No	No	No	No
Salmonella enterica subsp. *enterica* serovar Typhimurium LT2	GCA_000006945.2	No	No	No	Yes (4)	No	No	No	No	No
Actinobacillus equuli subsp. *equuli* strain 19392	GCA_000801145.1	No	No	No	No	No	No	No	No	No
Pasteurella multocida strain 20N	GCF_003061275.1	No	No	No	Yes (4)	No	No	No	No	No
Streptococcus equi subsp. *zooepidemicus*	GCF_015689455.1	No	No	No	No	Yes (5)	No	No	No	No
Escherichia coli strain 3_2_53FAA	GCA_000157115.2	No	No	Yes (6)	No	No	No	No	No	No
Klebsiella pneumoniae subsp. *pneumoniae* HS11286	GCA_000240185.2	No	No	No	No	No	No	No	No	Yes (7)
Corynebacterium pseudotuberculosis strain C231	GCF_000144675.2	No	No	No	Yes (5)	No	No	No	No	No
Staphylococcus aureus subsp. *aureus* strain NCTC 8325	GCF_000013425.1	No	No	No	No	Yes (4)	No	No	No	No
Pseudomonas aeruginosa strain PAO1	GCA_000006765.1	No	No	No	No	No	No	No	No	Yes (5)
Dietzia aerolata	GCF_014144865.1	No	No	No	No	Yes (5)	No	No	No	Yes (8)

		Match with Rhodo_Dlab			Match with Erm46_Dlab			Match with Erm51_Dlab		
**Species plasmids**	**RefSeq no.**	**FW**	**RV**	**Probe**	**FW**	**RV**	**Probe**	**FW**	**RV**	**Probe**
Rhodococcus ruber strain C1	CP044212.1	No	No	No	No	No	No	No	No	Yes (7)
	CP044213.1	No	No	Yes (10)	No	No	No	No	No	No
Rhodococcus qingshengii strain CS98	AP023173.1	No	No	No	No	No	No	No	No	Yes (6)
Rhodococcus pyridinivorans strain SB3094	NC_023144.1	No	No	No	No	No	No	No	No	Yes (7)
	NC_023145.1	No	No	No	No	No	No	No	No	No
Rhodococcus opacus strain 1CP	CP009112.1	No	No	No	Yes (3)	No	No	No	No	No
	CP009113.1	No	No	No	No	No	No	No	No	Yes (9)
Rhodococcus fascians strain D188	CP015236.1	No	No	No	No	No	No	No	No	Yes (8)
	CP015237.1	No	No	No	No	No	No	No	No	Yes (7)
Rhodococcus erythropolis strain R138	CP007256.1	No	No	No	No	No	No	No	No	No
	CP007257.1	No	No	No	No	No	No	No	No	Yes (6)
Mannheimia haemolytica strain USDA-ARS-USMARC-191	CP023045.1	No	No	No	No	No	No	No	No	No
Mycolicibacterium smegmatis strain FDAARGOS_679	CP054796.1	No	No	No	No	No	No	No	No	No
Salmonella enterica subsp. *enterica* serovar Typhimurium LT2	AE006471.2	No	No	No	No	No	No	Yes (4)	No	No
Klebsiella pneumoniae subsp. *pneumoniae* HS11286	NC_016838.1	No	No	No	No	No	No	No	No	No
	NC_016839.1	No	Yes (3)	No	No	No	No	No	No	No
	NC_016840.1	Yes (7)	No	No	No	No	No	No	No	No
	NC_016841.1	No	No	No	No	No	No	No	No	No
	NC_016846.1	No	No	No	No	No	No	No	No	Yes (9)
	NC_016847.1	No	No	No	No	No	No	No	No	No

FW represents forward oligo; RV represents the reverse oligo; in brackets no. of SNP difference between oligo/probe and reference genome;*multiple alignments in the genome.In a second assay, we looked at the in -silico exclusivity by aligning the three oligo sets to 23 non- *R. equi* reference genomes selected based on previous research[9,10], genetic proximity to *R. equi,* or presence in the equine respiratory tract (Table 3). Although the three oligo sets showed potential illegitimate alignments with some genomes, no amplification was predicted because the forward and reverse oligos never matched the same genomic element.

Figure S1. Standard curves for qPCR in mocking communities

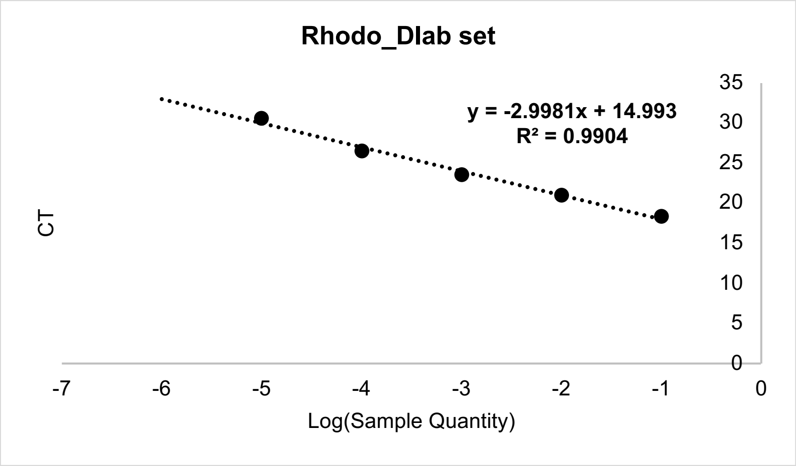

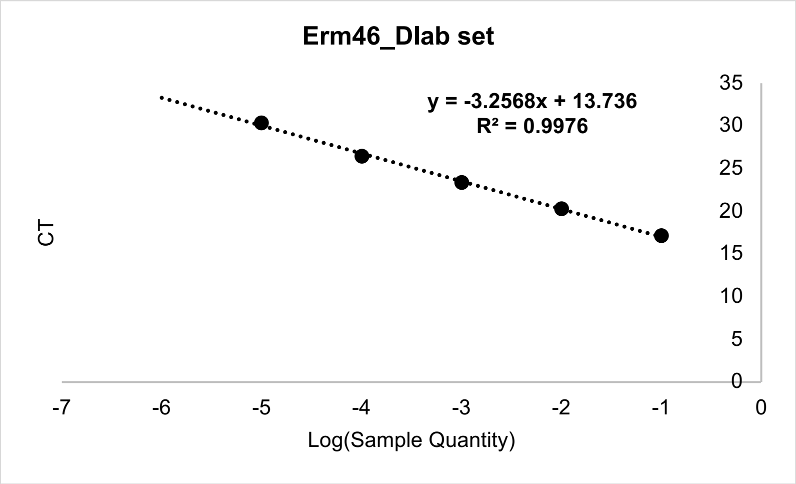

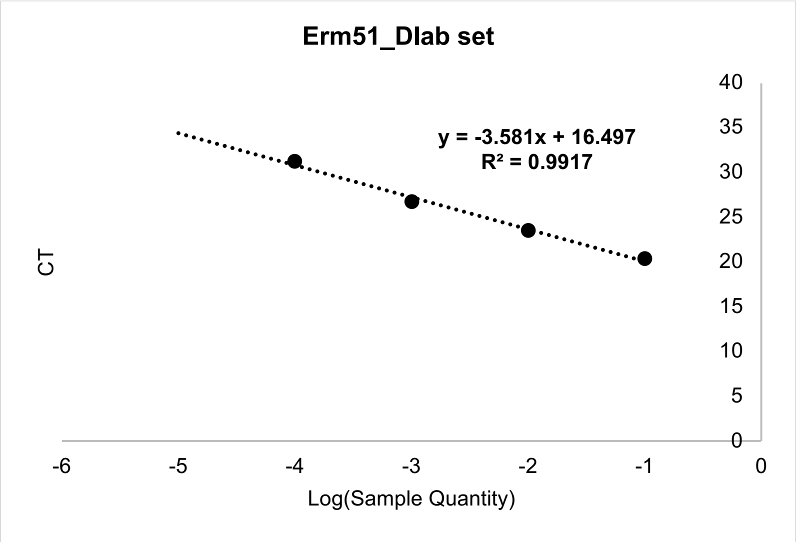

Table S3. *R. equi* clinical isolates tested in this study

Sample	Isolation source	Macrolide susceptibility	CT Rhodo_Dlab set	CT Erm46_Dlab set	CT Erm51_Dlab set
A15-04522	Dog	S	23.24	-	-
A15-08158	Dog	S	22.22	-	-
A17-41305	Horse	S	30.4	-	-
A21-34771	Dog	S	21.9	-	-
A21-38046	Racoon	S	21.01	-	-
A21-40530	Goat	S	24.26	-	-
A21-49275	Dog	S	33.91	-	-
A22-06434	Dog	S	21.71	-	-
A22-10736	Dog	S	21.87	-	-
A22-473838	Horse	S	26.37	-	-
A5-47992	Dog	S	20.27	-	-
A9-04013	Horse	S	37.17	-	-
